# Acipensins – Novel Antimicrobial Peptides from Leukocytes of the Russian Sturgeon Acipenser gueldenstaedtii

**Published:** 2014

**Authors:** O. V. Shamova, D. S. Orlov, S. V. Balandin, E. I. Shramova, E. V. Tsvetkova, P. V. Panteleev, Yu. F. Leonova, A. A. Tagaev, V. N. Kokryakov, T. V. Ovchinnikova

**Affiliations:** Institute of Experimental Medicine, Northwest Branch of the Russian Academy of Medical Sciences, Academician Pavlov Street, 12, Saint-Petersburg 197376, Russia; Shemyakin and Ovchinnikov Institute of Bioorganic Chemistry, Russian Academy of Sciences, Miklukho-Maklaya Street, 16/10, Moscow 117997, Russia; Saint-Petersburg State University, Universitetskaya Embankment, 7/9, Saint-Petersburg 199034, Russia; Moscow Institute of Physics and Technology (State University), Department of Physicochemical Biology and Biotechnology, Institutskii Pereulok, 9, Dolgoprudny 141700, Moscow Region, Russia

**Keywords:** innate immunity, antimicrobial peptides, sturgeon leukocytes, histone H2A derivatives, acipensin

## Abstract

Antimicrobial peptides (AMPs) play an important role in the innate defense mechanisms in humans and animals. We have isolated and studied a set of antimicrobial peptides from leukocytes of the Russian sturgeon* Acipenser gueldenstaedtii *belonging to a subclass of chondrosteans, an ancient group of bony fish. Structural analysis of the isolated peptides, designated as acipensins (Ac), revealed in leukocytes of the Russian sturgeon six novel peptides with molecular masses of 5336.2 Da, 3803.0 Da, 5173.0 Da, 4777.5 Da, 5449.4 Da, and 2740.2 Da, designated as Ac1–Ac6, respectively. Complete primary structures of all the isolated peptides were determined, and the biological activities of three major components – Ac1, Ac2, and Ac6 – were examined. The peptides Ac1, Ac2, Ac3, Ac4, and Ac5 were found to be the N-terminal acetylated fragments 1–0, 1–5, 1–9, 1–4, and 1–1 of the histone H2A, respectively, while Ac6 was shown to be the 62–5 fragment of the histone H2A. The peptides Ac1 and Ac2 displayed potent antimicrobial activity towards Gram-negative and Gram-positive bacteria (*Escherichia coli *ML35p, *Listeria monocytogenes *EGD, MRSA ATCC 33591) and the fungus *Candida albicans *820, while Ac6 proved effective only against Gram-negative bacteria. The efficacy of Ac 1 and Ac2 towards the fungus and MRSA was reduced upon an increase in the ionic strength of the solution. Ac1, Ac2, and Ac6, at concentrations close to their minimum inhibitory concentrations, enhanced the permeability of the *E.coli *ML35p outer membrane to the chromogenic marker, but they did not affect appreciably the permeability of the bacterial inner membrane in comparison with a potent pore-forming peptide, protegrin 1. Ac1, Ac2, and Ac6 revealed no hemolytic activity against human erythrocytes at concentrations of 1 to 40 μM and had no cytotoxic effect (1 to 20 μM) on K-562 and U-937 cells *in vitro*. Our findings suggest that histone-derived peptides serve as important anti-infective host defense molecules.

## INTRODUCTION

Antimicrobial peptides (AMPs) of the innate immune system play an essential role in the anti-infective protection of humans and animals [1]. These molecular factors of innate immunity are of particular importance in providing protective functions to lower vertebrates (fish, amphibians), because the system of adaptive immunity in poikilothermic animals cannot ensure a sufficiently rapid and effective response (antibody formation) to infection at a low temperature in the environment. Therefore, an investigation of the antimicrobial peptides of fish phagocytes and mucous coats is important for explaning the biological role of this group of physiologically active substances in anti-infective immunity.

To date, the AMPs (defensins, cathelicidins, etc) of phagocytes and barrier epithelium of various mammals, birds, and amphibians have been described. Information on fish AMPs remains limited and mostly relates to investigation of the compounds isolated from the mucus, skin, gills, kidneys, spleen, and gastrointestinal tract [2]. For example, pleurocidins [3, 4], which are a group of linear antimicrobial peptides with an α-helix conformation and a positive charge of the molecule, were isolated from the mucus of the* Pleuronectes americanus *flounder. A peptide called pardaxin was isolated from the mucous secretions of another flounder species, *Pardachirus marmoratus* [5]. The peptide hepcidin containing four intramolecular disulfide bridges was isolated from the gills of the hybrid striped bass [6]. Later, hepcidins were found in other fishes. An antimicrobial peptide, misgurin, with broad-spectrum microbicidal activity was obtained from the skin mucus of the loach (mudfish) *Misgurnus anguillicaudatus *[7].

Peptides of the α-defensin family have not yet been found in fish. However, in recent years, data on β-defensins of bony fish have appeared: these peptides were found in epithelial cells of the digestive tract, gills, and spleen of the Chinese perch *Siniperca chuatsi* [8], in the liver of the orange-spotted grouper *Epinephelus coioides *[9], and in the skin and gills of the carp *Cyprinus carpio *L. [10]. Peptides of the cathelicidin family have been found in the rainbow trout and other salmon and cod species [11-15].

Peptides that are histone derivatives have been found in the skin and mucous coats of some fishes. For example, antimicrobial peptides that are N-terminal parts of histone H2A were isolated from the skin mucus of the Atlantic halibut (*Hippoglossus hippoglossus* L.) and the Amur catfish *Parasilurus asotus *and called hipposin [16] and parasin 1 [17], respectively. An antimicrobial protein (SAM) with a molecular weight of 20,734 Da that appeared to be histone H1 was isolated from the liver of the *Salmo salar *salmon [18].

Therefore, to date, a number of antimicrobial peptides have been described that were isolated from the mucous coats, skin, digestive tract, gills, liver, and spleen of fish. However, there is actually a scarcity of data on AMPs from fish blood leukocytes. Therefore, the aim of this study was to investigate the structural properties and biological activity of antimicrobial peptides from blood leukocytes of the Russian sturgeon* Acipenser gueldenstaedtii*, a member of the subclass Chondrostei, the oldest group of bony fish (Osteichthyes).

## EXPERIMENTAL

**Reagents**

We used acrylamide, N,N’-methylene-bis-acrylamide (Sigma, USA), urea, sodium chloride, tris(hydroxymethyl) aminomethane, MTT (3-[4,5-dimethyl-2-thiazolyl]- 2,5-diphenyl-2H-tetrazolium bromide), agarose, trypticase soy broth, trifluoroacetic acid, heptafluorobutyric acid, o-nitrophenyl-β-D-galactopyranoside (Sigma, USA); acetic acid (Vekton, Russia); media and sera for cell cultures (Biolot, Russia); Saburo medium (Pharmacotherapy Research Center, Russia); and enzymes and buffers for PCR and genetic engineering (Thermo Fisher Scientific, USA). Antimicrobial peptides were used as standards: chemically synthesized protegrin 1 kindly provided by Professor Robert Lehrer (University of California, Los Angeles, USA), human defensin HNP-1 and bactenecin 5 isolated from human and goat blood leukocytes, respectively, using the method described previously [19].

**Isolation and purification of antimicrobial peptides from leukocytes of the Russian sturgeon**

Blood from *A. gueldenstaedtii *sturgeons, caught in the Volga delta (Alexandrovskiy sturgeon plant), was stabilized by heparin and kept in vessels for 6 h for delamination, after which the buffy coat was pipetted off and washed twice with saline, followed by centrifugation at 400 g for 5 min. The resulting precipitate was homogenized. Extraction of proteins from the leukocyte-rich suspension was carried out in 20% acetic acid at 4 °C under magnetic stirring for 20 h. The homogenate was then centrifuged at 15,000 g for 1 h. The supernatant was collected and subjected to ultrafiltration through the YM-10 membrane using a device from Amicon (USA). The resulting material containing peptides and low-molecular-weight proteins with molecular weights under 10–12 kDa was concentrated to 1 mL by ultrafiltration through the YM-0.5 membrane and loaded onto an electrophoretic column (a sample was preliminarily added with urea to a concentration of 3 M) for separation of the proteins by preparative electrophoresis (EP). Preparative EP with continuous elution of proteins was performed in a 12.5% polyacrylamide gel (PAAG) in an acidic buffer system in the presence of urea [20] using a Bio-Rad cell (USA). Protein fractions eluted from the column were analyzed in the presence of sodium dodecyl sulfate [21] by analytical EP, which was carried out in PAAG plates on a Hoeffer device (USA). The solution absorbance of each fraction was measured at 280 nm, and the antimicrobial activity was determined. The fractions with antimicrobial activity were collected, and the peptides present in them were separated by means of several successive cycles of reverse phase high-performance liquid chromatography (RP-HPLC) on a Gold System instrument (Beckman, USA) using Vydac C-18 columns (4.6 × 250 mm; 10 × 250 mm, the diameter of sorbent particles was 5 μm), eluting peptides with the gradient of acetonitrile concentration using different counterions (0.1% trifluoroacetic acid or 0.13% heptafluorobutyric acid). The fractions obtained by RP HPLC were lyophilized by centrifugation under vacuum using a SpeedVac device (Savant, USA). Evaluation of the purity was performed by analytical EP, MALDI TOF mass spectrometry, and analytical RP HPLC. The protein concentration in the extracts from sturgeon leukocytes and in the purified peptide samples was determined by the Bradford method and the Wolf method using the following equation: peptide concentration (μg/mL) = (A_215_ – A_225_) × 144 [22].

**Evaluation of peptide antimicrobial activity by radial diffusion in agarose gels**

The antibiotic effect of the peptides isolated from leukocytes was measured by the method proposed by Lehrer *et al*. [23]. The antimicrobial activity of the samples towards a number of Gram-negative and Gram-positive bacteria, as well as one of the fungi, was determined. The following strains of bacteria were used: Gram-negative bacterium *Escherichia coli *ML- 35p, Gram-positive bacteria: *Listeria monocytogenes* EGD and MRSA ATCC 33591 (methicillin resistant* Staphylococcus aureus*), and fungus *Candida albicans* 820. The bacteria were grown on a solid medium containing a 3% trypticase soy broth (Sigma, USA); the fungi were grown in a medium containing 3% Saburo. When *E. coli *ML-35P was cultured, the medium was supplemented with 100 μg/mL ampicillin and the medium for MRSA ATCC 33591 was supplemented with 6 μg/mL oxacillin. Microorganism strains were kindly provided by Professor Robert Lehrer (University of California, Los Angeles, USA).

The microorganisms were cultured for 16–18 h in a medium containing a 3% trypticase soy broth (TSB) solution at 37 °C. Bacterial suspension aliquots were taken from the resulting culture, transferred to the fresh 3% TSB solution, and incubated at 37 °C for 2.5 h to obtain microorganisms in the logarithmic growth phase. The overnight culture was used in experiments with the fungus *C. albicans*. Microorganism suspensions were further centrifuged at 400 g for 10 min, the precipitate was washed twice with a 10 mM sodium phosphate buffer (PBS), pH 7.4, and re-suspended in 3 mL of the same buffer. To prepare agarose gels containing microorganisms, the volume of the suspension with 4 × 106 cells was calculated [23]. The number of bacterial cells was evaluated by measuring the suspension optical density at 620 nm (it was assumed that the optical density of 0.2 corresponds to 5 × 10^7^ CFU/mL). Another equation was used for fungi: the optical density of 1 at 450 nm corresponds to 2.86 × 10^7^ CFU/mL [23]. The calculated amount of microorganism suspension was added to 8 mL of a sterile 1% agarose solution in a 10 mM sodium phosphate buffer (in some experiments, 100 mM NaCl), pH 7.4, at a temperature of 43 °C. The resulting mixture was poured into a sterile plastic Petri dish with a diameter of 90 mm, wherein the mixture was solidified to form an agarose gel. Holes with a diameter of 2 mm were perforated in the agarose gels. Wells were filled with the analyzed samples (serial (two-fold) dilutions of the peptides in a 0.01% aqueous acetic acid solution and a control, 0.01% acetic acid without peptides) and incubated at 37 °C for 3 h. During incubation, the peptides diffused from the wells into the agarose gels. After completing incubation, a 1% agarose solution containing a 6% trypticase soy broth was applied to the agarose gel surface. The dishes were then incubated for more 20 h at 37 °C. To quantify the peptide antibiotic activity, the diameter of the microbial growth inhibition zone around the wells was measured, taking 0.1 mm as the unit, and 20 units were subtracted, which corresponded to the well diameter, from the measured value. The minimum inhibitory concentration (MIC) was determined for each peptide by plotting the dependence of the peptide antimicrobial activity on their concentration. The value obtained for the intersection of the plot of the linear regression of each peptide with the X axis (peptide concentration in μM) was taken as the MIC. Two parallel samples were tested in each experiment. Experiments were performed in triplicate, and the arithmetic mean of the obtained MIC ± standard deviation was calculated.

**Evaluation of the peptide’s effect on the permeability of the outer and cytoplasmic membranes of *E. coli *ML35p**

The *E. coli *ML35p strain used in this method is characterized by a lack of lactose permease (enzyme transporting lactose into the cell), with the synthesis of β-galactosidase in the bacterium cytoplasm being constitutive, not inducible as in most bacteria. Furthermore, the β-lactamase enzyme is present in the periplasmic space of *E. coli *ML35p [24]. The state of the cytoplasmic and outer membranes of *E. coli *ML35p was judged by their permeability to chromogenic markers, o-nitrophenyl- β-*D*-galactopyranoside (ONPG) and nitrocefin, which are substrates of β-galactosidase and β-lactamase, respectively. A modification of the previously described procedure was used [25]. If the medium surrounding the bacteria contains substrates of β-galactosidase or β-lactamase, the enzymatic reaction involving these substrates can occur only if they are able to penetrate through the bacterial membranes. If the outer and cytoplasmic membranes of the bacteria, under the action of some damaging agent, such as an antimicrobial peptide, become permeable to the substrates, then the chromogenic products of substrate hydrolysis by intracellular enzymes pass into the incubation medium. The optical density of the medium at 496 or 420 nm (absorption maximum of the chromogenic products of hydrolysis of nitrocefin or ONPG, respectively) increases, which enables monitoring, in real time, of the process of damage to the outer and cytoplasmic membranes of the bacteria by the antimicrobial agent. Sample composition (100 μL): 2.5 mM ONPG or 20 μM nitrocefin; 2.5 × 10^7^ CFU/mL of bacteria; 0.01 M Na-phosphate buffer, pH 7.4; 0.03% TSB; the peptides at a concentration that is equal to their minimum inhibitory concentration under these conditions and is measured by a standard colony count method. The controls contained equal volumes of the solvent (0.01% acetic acid) instead of preparations. The samples were added into the wells of a 96-well plate, and the solution absorbance was measured at 496 and 420 nm using a SpectraMax 250 spectrophotometer (Molecular Devices, USA) at 37 °C and under periodic shaking of the plates for 2 h. The data were processed with the Sigma Plot 11 software. The plots present the results of a typical experiment: each point is the average of two values obtained for duplicate samples. The experiments were performed in triplicate, and the curve pattern was similar for all three series.

**Analysis of peptide hemolytic activity**

Blood from healthy donors was collected in plastic tubes, using heparin as an anticoagulant and centrifuged at 250 g, 4 °C, for 10 min. The supernatant was removed, and the pellet was added with 10 mL of buffered saline (PBS), pH 7.4, with 4 mM EDTA and centrifuged at 250 g, 4 °C, for 10 min. The precipitate was washed 3 times with PBS using centrifugation as described above. 280 μL of the erythrocyte precipitate (the precipitate was assumed to be 100% red blood cells) was adjusted to 10 mL with cold PBS to obtain a 2.8% suspension. The analyzed samples were added with 27 μL of the erythrocyte suspension and 3 μL of the test peptide at various concentrations in PBS. To prepare a positive control (100% erythrocyte lysis), 27 μL of the erythrocyte solution was added with 3 μL of a surfactant (10% Triton X-100). To prepare a negative control (0% erythrocyte lysis), 27 μL of the erythrocyte solution was added with 3 μL of PBS. Samples in triplicate were incubated at 37 °C for 30 min, and the reaction was terminated by adding 75 μL of ice-cold PBS. The samples were centrifuged at 5,000 g, 4 °C, for 4 min, and the supernatant was collected and added to the wells of a 96-well plate (Corning, USA). The sample’s optical densities at 540 nm were measured on a SpectraMax 250 spectrophotometer (Molecular Devices, USA). The erythrocyte hemolysis parameters were calculated as a percentage according to the equation:

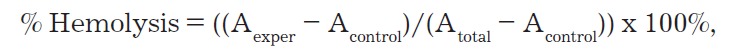



where A_exper_ and A_control_ are the supernatant absorbance obtained for treated with the peptides and untreated (0% lysis) erythrocytes, respectively, and A_total_ is the supernatant absorbance for red blood cells treated with Triton X-100 (100% lysis).

**MTT test**

The effect of the peptides on cell viability was examined by the MTT assay [26]. This test is based on the ability of dehydrogenases from living cells to reduce colorless forms of 3-(4,5-dimethylthiazol-2-yl)-2,5-diphenyltetrazolium bromide (MTT reagent) or 3-(4,5-dimethylthiazol- 2-yl)-2,5-diphenyl-2H-tetrazolium bromide to a blue formazan crystal. The cultured cell lines K-562 (human erythroleukemia cells) and U-937 (human histiocytic lymphoma cells) were used in the experiments. The cell suspensions were placed into the wells of 96- well plates (Orange Scientific, Belgium) in a RPMI-1640 medium, 20,000 cells per well. The cells were added with 10 μL of a peptide solution (in RPMI-1640 medium) at various concentrations in quadruplicate. Control samples were added with 10 μL of the medium instead of the peptides. The plates were then placed in a CO_2_ incubator for 20 h. Three hours before the end of incubation, the plate wells were added with 10 μL of the MTT solution (5 mg/mL in PBS). After incubation, the wells were added with 100 μL of isopropanol with 0.04 M HCI, stirred, and the optical density of the solution in plate wells was measured at 540 nm (subtracting the absorbance at 690 nm as a background) on a Spectra Max 250 spectrophotometer (Molecular Devices, USA). The significance of the differences between groups was evaluated using the Wilcoxon-Mann-Whitney U-test. In all calculations, the 95% confidence level (P < 0.05) was considered as significant.

**Mass Spectrometry**

The molecular weights of the isolated peptides were determined on a Reflect III MALDI-TOF mass spectrometer (Bruker, Germany) equipped with a UV-laser with a wavelength of 336 nm. 2,5-dihydroxybenzoic acid (Sigma, Germany) in 20% acetonitrile and 0.1% TFA at a concentration of 10 mg/mL was used as a matrix.

**Determination of the N-terminal amino acid sequence**

The amino acid sequence was determined by using a Procise cLC 491 protein sequencing system (Applied Biosystems, USA). The phenylthiohydantoin derivatives of amino acid residues were identified on a 120A PTH analyzer (Applied Biosystems, USA).

**Deblocking of N-terminal amino acid residues**

Deacylation of the N-terminal acetylserine residue was performed according to the procedure in [27] on an inert carrier, Immobilon, used for automatic protein sequencing. A peptide sample was applied to Immobilon, placed in a 500 μL test tube, damped with 30 μL of 25% TFA solution, and then incubated in a sealed test tube at 45 °C for 4 min. Peptide-coated Immobilon was dried in an open test tube at 20 °C for 5 min and at 45 °C for another 10 min and then incubated in a sealed test tube at 45 °C for 72 h. The resulting sample was used for automated sequencing of the peptide.

**Isolation of genomic DNA**

Sturgeon tissue fragments were incubated in the TNES buffer (10 mM Tris-HCl, pH 7.5; 400 mM NaCl; 100 mM EDTA, 0.6% SDS) with 10 mg/mL proteinase K at 55 ° C for 16 h. After centrifugation at 12,000 g for 10 min, the supernatant was added with a 0.25 volume of 5M NaCl and DNA was precipitated by adding one volume of 95% ethanol. The precipitate was washed with 80% ethanol, dried in an open test tube, and dissolved in water. Then, a single purification of DNA was performed with the phenol : chloroform mixture (1 : 1, v/v); the remaining phenol was extracted with an equal volume of chloroform, and DNA was precipitated with 95% ethanol.

**Amplification of nucleotide sequences**

To amplify an internal region of the sturgeon histone H2A gene, degenerate primers №1 (ATGTGTGGACG(A,C)GG(C,T)AA(A,G)AC(A,C,T) GG) and №2 (GTCTTCTTGGG(C,G)AG(C,T)AG(C,T) AC(G,T)GCC) were used. PCR was performed with a stepwise reduction in temperature at the stage of primer annealing: 94 °C – 1 min; 5 × (94 °C – 30 s, 61 °C – 40 s, 68 °C – 60); 5 × (94 °C – 30 s, 58 °C – 40 s, 68 °C – 60); 30 × (94 °C – 30 s, 55 °C – 40 s, 68 °C – 60 s).

Prior to inverse PCR, the sturgeon genomic DNA was treated with BglII restrictase. Composition of the reaction mixture: 1 × buffer O (Thermo Fisher Scientific), 50 ng/μL DNA, 40 units of BglII (Thermo Fisher Scientific). The reaction was conducted in a volume of 50 μL at 37 °C for 16 h. The reaction products were diluted 25 times with water (2 ng/μL of DNA), followed by the ligation reaction conducted in the presence of 10 mM ATP at 4 °C for 16 h. Then, DNA was precipitated with 95% ethanol: the precipitate was dissolved in water and used as a template for inverse PCR.

Inverse PCR was conducted using two primer pairs in two stages. At the first stage, primers №3 (GAGCACAGCGGCCAGATAGA) and №4 (CTGAAATCCT GGAGCTGGC) and the following amplification program were used: 94 °C – 5 min; 35 × (94 °C – 30 s, 58 °C – 60 s, 72 °C – 120 s). The resulting product was diluted with water (1:100) and used as a template for nested PCR with primers №5 (CACGCTGGGCATAGTTTCC) and №6 (GAATCATCCCGCGTCACCTG) under the following conditions: 94 °C – 5 min; 35 × (94 °C – 30 s, 62 °C – 60 s, 72 °C – 120 s).

**Cloning and sequencing of PCR products**

PCR products were eluted from low-melting-point agarose and ligated into the pGEM-T vector at sticky T/A ends (Promega, USA). Manipulations with recombinant DNA were performed according to the reference procedures [28]. Competent cells of the *Escherichia coli *DH-10B strain (Life Technologies, USA) were used for transformation. The plasmid DNA was isolated by alkaline lysis. DNA sequencing was performed using the ABI PRISM BigDye Terminator v. 3.1 reagent kit, followed by an analysis of the reaction products on a 3730 DNA Analyzer automatic sequencer (Applied Biosystems, USA).

**Statistical analysis of results**

Data obtained in the study of peptide antimicrobial activity are presented as an arithmetic mean + standard deviation. The arithmetic mean was calculated based on three independent experiments, each of which was performed in duplicate. The data were processed with the Statistica 6 software.

## RESULTS

**Structural study of AMPs from leukocytes of the Russian sturgeon**

To isolate the antimicrobial peptides from Russian sturgeon leukocytes, a procedure for isolation and purification was used that involved acid extraction of proteins and peptides from leukocytes, ultrafiltration, preparative EP, and reverse-phase high-performance liquid chromatography [29]. The use of this set of methods yielded six purified AMPs with molecular weights of 5,448.8 Da, 5,336.0 Da, 5,174.0 Da, 4,777.6 Da, 3,804.0Da, and 2,741.7 Da determined using MALDI TOF mass spectrometry, with the three peptides with molecular weights of 5,336.0 Da, 3,804.0 Da, and 2,741.7 Da accounting for the predominant peptide fractions. The described isolation procedure was repeated several times using the blood leukocytes of sturgeons that were caught at different times and were of different ages and genders, with each cycle of isolation resulting in a similar spectrum of the antimicrobial peptides. In some experiments, during preparation of the leukocyte-rich suspension and extraction, the serine protease inhibitor, phenylmethylsulfonyl fluoride, was used which did not lead to a significant change in the spectrum of the isolated antimicrobial peptides. The objectives of this study included the structural analysis of the selected peptides and investigation of their biological activity.

A partial N-terminal amino acid sequence of the isolated peptides was established by automated microsequencing. In some cases, sequencing was possible after the chemical reaction of deblocking of the N-terminal amino acid residues. Removal of the acetyl group of the N-terminal acetylserine residue was carried out in an acidic medium using a 25% solution of TFA. After the removal of the acetyl protection, the N-terminal amino group was easily modified with phenylisothiocyanate, which allowed stepwise degradation of the peptides by the Edman method using a Procise cLC 491 protein sequencing system and identification of the obtained phenylthiohydantoin derivatives of the amino acid residues.

The analysis of the N-terminal peptide sequences from the Russian sturgeon *Acipenser guldenstaedtii*, which we named acipensins 1–5, using the BLAST software (http://blast.ncbi.nlm.nih.gov/Blast.cgi) demonstrated that they were the N-terminal fragments of a histone of the H2A family, while another peptide, acipensin 6, was a fragment of the central part of the histone molecule of the same family. These AMPs constitute the dominant part of acid-soluble peptides with antimicrobial activity from sturgeon leukocytes.

**Fig. 1:**
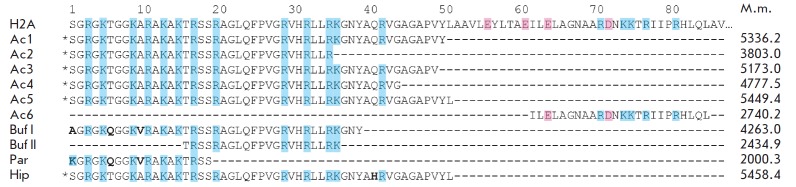
The structure of acipensins and related AMPs. Histone H2A N-terminal amino acid sequence (*A. gueldenstaedtii*) and Ac1–Ac6 acipensins are presented. Buf I and Buf II – buforins I and II (*Bufo bufo gargarizans*); Par – parasin 1 (*Parasilurus asotus*); Hip – hipposin (*Hippoglossus hippoglossus *L.). The asterisk (*) denotes acetylated N-terminal amino acid residues. M.m. – calculated molecular weights of AMPs, Da. Lys and Arg residues are highlighted with blue; Asp and Glu residues are highlighted with pink. The single amino acid differences between AMP sequences and sturgeon histone H2A are shown in bold

To determine the complete primary structure of the acipensins, cloning and sequencing of encoding nucleotide sequences was performed. It is known that the genes of canonical histones, which are most widely present in eukaryotic cells, lack introns, and that their mRNAs lack the 3’-terminal polyA sequence [30]. Only mRNAs of specialized histone variants constituting a minor fraction of these proteins are subjected to polyadenylation. Another feature of the canonical histones is the presence of multiple copies of their genes in the genome. All this makes it preferable to use genomic DNA as a template for the amplification of nucleotide sequences encoding histones. Based on the assumption that the isolated AMPs were products of the partial proteolysis of the dominant fraction of the sturgeon histone H2A, it was decided to amplify a region of the genomic DNA encoding this histone. Due to the low evolutionary conservation of the untranslated regions flanking this site, the experiment was carried out in two stages. The internal part of the gene with degenerate primers selected for conserved segments of the translated region were first amplified. Cloning and sequencing of the resulting amplicon about 300 bp in length enabled selection of the structure of primers for inverse PCR that was used to amplify the nucleotide sequence of the regions immediately adjacent to the fragment sequenced at the first stage. The amplicon size in this case was about 400 bp. The data obtained by DNA sequencing (GenBank KP059880) in combination with the data of the N-terminal amino acid analysis and mass spectrometry allowed us to determine the complete amino acid sequence of acipensins 1–6 (*Fig. 1*). It was found that Ac1, Ac2, Ac3, Ac4, and Ac5 are the N-terminal acetylated fragments 1–50, 1–35, 1–49, 1–44, and 1–51 of histone H2A, respectively, and Ac6 is the fragment 62–85 of histone H2A. The structural analysis of acipensins 1–6 showed the presence of peptides with calculated molecular weights of 5,336.2 Da, 3,803.0 Da, 5,173.0 Da, 4,777.5 Da, 5,449.4 Da, and 2,740.2 Da, respectively, in sturgeon leukocytes. The calculated molecular weights of acipensins 1–6 were in good agreement with the experimental data of massspectrometry analysis (5,336.0 Da, 3,804.0 Da, 5,174.0 Da, 4,777.6, 5,448.8 Da, and 2,741.7 Da, respectively).

**Analysis of acipensin antimicrobial activity**

Antimicrobial activity is believed to be a central functional property of AMPs. The peptides described in the literature (defensins, cathelicidins, etc) have it to different extents: the mechanism of their antibacterial action is diverse. The antimicrobial activity of three major fractions of acipensins (Ac1, Ac2, Ac6) was evaluated by radial diffusion, with experiments being conducted under various conditions: in a medium containing only a 10 mM sodium phosphate buffer with no added salts and in the same medium but with 100 mM sodium chloride (close to the physiological concentration of sodium chloride). This approach aimed at evaluating the impact of an increase in the solution’s ionic strength on the efficiency of the antimicrobial activity of peptides was used in many experimental studies of the antimicrobial properties of natural AMPs described in the literature, and its use enables a comparison of the activity of the isolated peptides with the antibiotic effects of other AMPs. The results of the analysis of the acipensin’s antimicrobial activity against bacteria *Esherichia coli *ML-35p, *Listeria monocytogenes *EGD, methicillin- resistant *Staphylococcus aureus *(MRSA) ATCC 33591, and the fungus *Candida albicans *820 are presented in Table 1. The activity of acipensins was studied in comparison with three other AMPs with a different structure and mechanism of antimicrobial action: porcine protegrin 1, human alpha-defensin HNP-1, and goat bactenecin ChBac5. Alpha-defensins are the main antimicrobial peptides, along with cathelicidin LL-37, of human neutrophilic granulocytes. Proline-rich bactenecins are the dominant family of the AMPs of goat and sheep neutrophils. Protegrin 1 (PG-1) of porcine leukocytes, which has a beta-hairpin conformation, is one of the most active peptides, described to date, of animal leukocytes that exhibits a broad spectrum of antimicrobial activity based on its ability to damage the membranes of microorganisms.

**Table 1:** Antimicrobial activity of acipensins Ac1, Ac2, and Ac6*

	Minimum Inhibitory Concentration, μM							
	E.coli ML35p		Listeria monocytogenes EGD		MRSA ATCC 33591		Candida albicans 820	
	without NaCl	100 mM NaCl	without NaCl	100 mM NaCl	without NaCl	100 mM NaCl	without NaCl	100 mM NaCl
Ac1	0.7 ± 0.1	0.4 ± 0.1	1.1 ± 0.2	2.3 ± 0.4	0.9 ± 0.2	> 40	1 ± 0.2	> 40
Ac2	0.3 ± 0.1	0.1 ± 0.2	1.0 ± 0.2	2.7 ± 0.3	0.6 ± 0.1	> 40	0.9 ± 0.1	> 40
Ac6	2.5 ± 0.3	> 40	> 40	> 40	> 40	> 40	> 40	> 40
HNP-1	0.8 ± 0.1	> 50	1.0 ± 0.3	1.1 ± 0.2	1.7 ± 0.3	> 50	2.71± 0.4	> 50
PG-1	0.2 ± 0.1	0.2 ± 0.1	0.3 ± 0.05	0.3 ± 0.1	0.4 ± 0.1	0.4 ± 0.2	0.4 ± 0.1	1.2 ± 0.4
ChBac5	0.4 ± 0.1	0.3 ± 0.1	0.6 ± 0.1	1.5 ± 0.7	0.8 ± 0.3	> 40	0.9 ± 0.2	> 40

* Data are presented as the minimum inhibitory concentration of peptides in μM; the peptides were incubated with microorganisms in a 10 mM sodium phosphate buffer, pH 7.4, in one case, and in a 10 mM sodium phosphate buffer, pH 7.4, containing 100 mM NaCl, in another case. The comparison peptides were porcine protegrin 1 (PG-1), human alphadefensin HNP-1, and goat bactenecin ChBac5.

As seen from the Table, in a medium with a low ionic strength, acipensins 1 and 2 demonstrate a broad spectrum of action and exhibit a high antimicrobial activity against both Gram-negative and Gram-positive bacteria, as well as the fungus. However, the spectrum of Ac1 and Ac2 activity changes with increasing medium ionic strength: the efficiency of their effect against the Gram-positive bacterium MRSA ATCC 33591 and the fungus *Candida albicans *820 is significantly reduced. Ac6 demonstrates an antimicrobial effect only against the Gram-negative bacterium *E.coli *in a low-ionicstrength medium. The antimicrobial effect of Ac1 and Ac2 differs from that of membrane-active PG-1 and is similar to the effects of bactenecin ChBac5. Bactenecins are known to exhibit antibacterial action mainly against Gram-negative bacteria, and this action is exerted without significant damage to the bacterial membranes and is directed against intracellular targets [14]. Although acipensins do not have a structural similarity with proline rich bactenecins, it may be assumed that the prime targets of their antibacterial action, similarly to bactenecins, are not bacterial membranes. Investigation of the effects of Ac1, Ac2, and Ac6 on the barrier function of bacterial membranes was the next objective of this study.

**The effect of acipensins on the permeability of the outer and cytoplasmic membranes of* E.coli *ML35p to chromogenic markers**

**Fig. 2:**
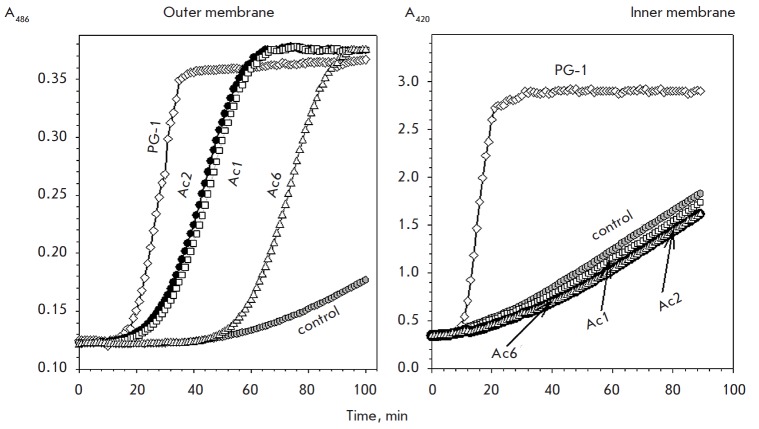
Kinetics of the changes in the *E.coli *ML35p membrane permeability to chromogenic markers under the action of antimicrobial peptides. X axis – time of incubation of peptides and bacteria, min. Y axis – optical density of a solution containing chromogenic markers: the nitrocefin hydrolysis product (left panel displaying the outer membrane permeability) and the ONPG hydrolysis product – o-nitrophenol (right panel displaying the inner membrane permeability). The peptides were applied at concentrations equal to their MICs for *E.coli *ML35p under similar conditions

*Figure *2 presents the kinetics of the action of acipensins 1, 2, and 6 (at a concentration equal to their minimum inhibitory concentration (MIC) against this bacterium) on the outer and inner (cytoplasmic) membranes of *E.coli *ML35p. The membrane-active peptide PG-1 was used as a standard. Acipensins 1 and 2 have a distinct effect on the permeability of the outer membrane of the bacterium to a chromogenic marker, nitrocefin (as evidenced by an increase in the solution’s optical density due to the appearance of a colored product of the nitrocefin cleavage (see Experimental section)), although to a lesser extent than PG-1. An increase in the outer membrane permeability was observed 15–29 min after the addition of Ac1 and Ac2 to bacteria and 50 min in the case of Ac6. However, an action of all three studied acipensins on the cytoplasmic membrane of* E.coli *ML35p, evaluated by this method, was hardly observed 90 min after the start of the experiment: the membrane permeability to the chromogenic marker ONPG was unchanged compared to the control values (indicators in samples without peptides). Therefore, these data suggest that the main target of acipensins at concentrations close to the MIC are not the bacterial membranes but intracellular components.

Apart from the antimicrobial activity, most AMPs are known to exhibit a variety of effects on the host cells, including a cytotoxic effect, causing cell death. We investigated the possibility of acipensin’s toxic effect on cells of a macroorganism.

**Acipensin cytotoxic activity against human cells *in vitro***

**Fig. 3:**
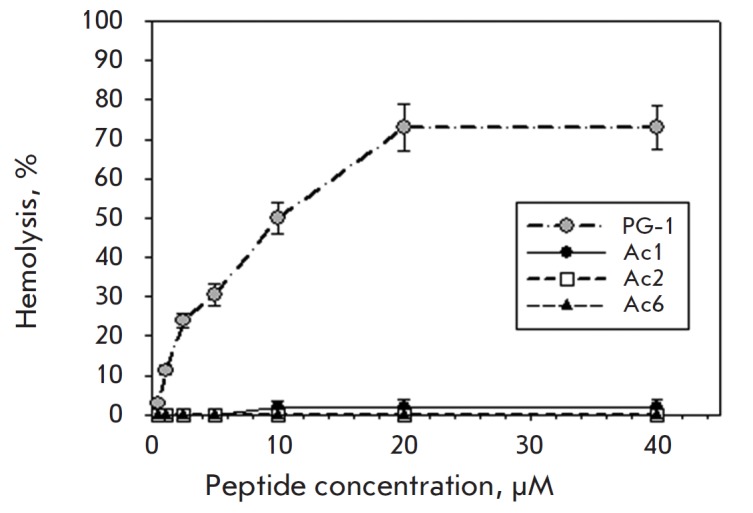
Hemolytic activity of acipensins. X axis – peptide concentration, μM. Y axis –percentage of erythrocyte hemolysis calculated by the following equation: % Hemolysis = ((A_exper_ –A_control_)/(A_total_ –A_control_)) x 100%, where A_exper_ and A_control_ are the supernatant absorbance obtained for treated and untreated red cells, respectively, and A_total_ is the supernatant absorbance for red cells treated with 1% Triton X-100. A strong hemolytic activity of the peptide PG-1 was used as a positive control

As in most published studies on the cytotoxic activity of various AMPs, human cells are the main subject of the investigation of this peptide’s activity, because natural AMPs are considered as promising prototypes of new drugs for use in medicine, and elucidation of the toxicity of these compounds, especially against human cells, is of great importance. We investigated the hemolytic activity of acipensins 1, 2, and 6 against human erythrocytes. Erythrocyte hemolysis was found not to be observed for the studied peptides at concentrations of 1–40 μM (*Fig. 3*). As in previous experiments, PG-1, which has a high hemolytic activity contrary to that of sturgeon peptides, was used as a positive control. A study of the acipensin effect on the cultured human cell lines K-562 (human erythroleukemia cells) and U-937 (human histiocytic lymphoma cells) revealed that the peptides at concentrations of 1–20 μM did not exert toxic effects on the target cells: the proportion of viable cells after their incubation with each of the studied acipensins for 20 h did not differ from the proportion of viable cells in the control samples.

## DISCUSSION

The discovery, in fish and amphibians, of cationic peptides that exhibit antimicrobial activity and are histone fragments allowed a number of researchers to suggest that these histone derivatives might have a non-nuclear localization and function as antimicrobial protectors [18, 32, 33]. This hypothesis is supported by data on the detection of histone H1 in the cytosol of human intestinal villus cells [34]. In mice, H1 was found on the macrophage surface, where it acts as a thyroglobulin receptor [35]. Histones H1 (MUMP-1 and MUMP-2) and H2B (MUMP-3) were isolated from the granular fraction of mouse macrophages [36]. In addition, histone H1 was found on the surface of mouse neurons [37], human monocytes [38], and the nonspecific cytotoxic cells of channel catfish (similar to natural killer cells of mammals) [39]. The importance of histones in the course of immune processes became apparent when information about their involvement in the functioning of neutrophil extracellular traps (NETs) emerged [40, 41]. The formation of these structures, first identified in 2004, is the third, along with phagocytosis and secretion of antimicrobial compounds, mechanism of neutrophil killing activity [42]. Extracellular traps are formed during NET osis (controlled cell death, significantly different from necrosis and apoptosis) and are a decondensed chromatin network that includes antimicrobial factors of both granular (proteases, AMPs) and nuclear (histones and products of their partial proteolysis) origin. Extracellular traps ensure capture and destruction of pathogenic microorganisms that, for some reason, cannot be neutralized by phagocytosis. Due to the structuring role of DNA, diffusion of antimicrobial factors from the trap is slowed, which enables achievement of high local concentrations of these substances and a reduction in their harmful effect on healthy tissues.

The obtained data on the structural and functional properties of antimicrobial peptides from leukocytes of the Russian sturgeon, acipensins, as histone H2A derivatives confirm the hypothesis that during evolution, derivatives of proteins that usually have a nuclear localization might be selected as endogenous antibiotic peptides in separate groups of animals. This demonstrates a surprising variety of structural AMP families among members of different taxa of the animal world. The most common group of AMPs is known to be members of the defensin family, whose functional activity is realized in phagocytes (neutrophils and macrophages of vertebrates, and amoebocytes and coelomocytes of invertebrates) and at the level of barrier epithelia of the integument and mucosae of animals. However, peptides of the defensin family were not found in sturgeon leukocytes. Defensins are also absent in the phagocytes of certain mammalian species (mouse, cat, dog, sheep and goat). In these species, the function of molecules inactivating phagocytized microbes is performed by peptides of the cathelicidin family (bactenecins, protegrins, etc). The peculiarity of the species pattern of the antimicrobial peptides of Russian sturgeon leukocytes is that they are dominated by peptides, histone H2A derivatives, that had not previously been found in fish phagocytes.

Acipensins have a broad spectrum of antimicrobial action like other, currently known histone H2A derivatives: buforin, parasin, hipposin, abhizin (peptide from the Haliotis mollusc) [16, 17, 31, 43]. Unlike parasin and buforin 1, acipensins 1–5 are molecules with an acetylated N-terminus. The hipposin molecule has the same property [16]. Acetylation of the N-terminus was found not to be necessary for the exhibition of antimicrobial activity, because synthetic non-acetylated hipposin turns out to be active [16].

The observed decrease in the antimicrobial activity of acipensins with an increase in the solution ionic strength is characteristic of many AMPs described in the literature: e.g., human alpha-defensins and goat bactenecins. The antimicrobial activity of defensins is known to be able to recover in media containing physiological concentrations of sodium chloride, which results from synergistic action with other AMPs of human neutrophils, in particular, cathelicidin LL-37 [44]. It may be assumed that, along with the constitutively synthesized acipensins, sturgeon leukocytes contain inducible antimicrobial factors whose synthesis is increased during the infectious process, and that can act together with acipensins, which increases the efficiency of the antimicrobial action of AMPs.

The data on the low antimicrobial activity of Ac6 compared to that of Ac1 and Ac2 suggest that these are N-terminal derivatives of histone H2A that play a key role in anti-infective protection.

The mechanisms of action of AMP derivatives of histone H2A described in the literature are slightly different: the main mechanism of antimicrobial action of buforin 2 is associated with its ability to penetrate into bacterial cells without significant damage to their membranes and to interact with nucleic acids, which leads to inhibition of vital processes in microbial cells and to their death [45, 46]. However, another peptide, parasin, exerts a pronounced damaging effect on the bacterial membranes [47]. A synthetic analog of hipposin was demonstrated also to have the ability to increase the permeability of bacterial membranes (*E. coli* ATCC 25922) [48]. Although acipensins have a significant structural similarity to hipposin, no appreciable increase in the permeability of the cytoplasmic membrane of *E. coli *ML35p under the action of acipensins was observed in our experiments. On the one hand, it may be assumed that the difference in the results is related to the fact that other bacterial strains and permeability markers were used in our study. On the other hand, the decisive role is probably played by the peptide concentration. For example, the dual mode of action [49] was established for proline-rich peptides, in particular bactenecins: at concentrations close to the MIC, the peptides did not have a damaging effect on the membranes, their effects were associated with the impact on intracellular targets, while at concentrations above the MIC, these AMPs, in addition to the inhibition of intracellular processes, disturbed the structural integrity of the membrane. Because concentrations close to the MIC were used in experiments on the evaluation of the acipensin effect on the permeability of bacterial membranes, we may assume that acipensins at higher concentrations, like bactenecin and hipposin, can also disturb the barrier function of bacterial membranes. Finally, it cannot be excluded that the observed discrepancy in the results may be due to those few amino acid substitutions that distinguish Ac1 and Ac2 from the hipposin analog used in [48]. A more detailed investigation of the effect of acipensins on bacterial membranes will be conducted in our future studies using recombinant acipensin analogs.

Similarly to other natural AMP derivatives of histone H2A, all three studied acipensins (Ac1, Ac2, and Ac6) exhibited no significant cytotoxic activity against the cultured human cells. Further investigation of the interaction of acipensins and their structural analogs with the cells will establish whether they possess the potential to translocate across the eukaryotic cell membranes, as it was demonstrated for buforins [46]. Having similar properties opens prospects for the practical application of the peptides in antitumor therapy as vectors for the delivery of drugs into malignant cells.

## CONCLUSIONS

A set of antimicrobial peptides called acipensins that are histone H2A fragments were for the first time isolated from leukocytes of the Russian sturgeon *A. gueldenstadti*. These peptides have a broad spectrum of antibacterial activity and do not exhibit toxic properties towards host cells. The obtained data contribute to the development of ideas regarding the evolution of the molecular factors of innate immunity and support the assumption of the biological role of histones as protective molecules involved in the implementation of the anti-infective function of the immune system.

## Abbreviations

Ac: acipensin
AMP: antimicrobial peptides
CFU: colony forming units
EDTA: ethylenediaminetetraacetic acid
HNP: human neutrophil peptide (alpha-defensin)
MIC: minimum inhibitory concentration
MRSA: methicillin resistant Staphylococcus aureus
ONPG: ortho-nitrophenyl β-D-galactopyranoside
PAGE: polyacrylamide gel electrophoresis
PBS: phosphate buffered saline
PCR: polymerase chain reaction
PG-1: protegrin 1
SDS: sodium dodecyl sulfate
TFA: trifluoroacetic acid
TSB: trypticase soy broth
MALDI-TOF-MS: matrix-assisted laser desorption ionization time of flight mass spectrometry
Tris: tris (hydroxymethyl) aminomethane
